# Supplementary material for: Are carbon emissions trading and green financial instruments synergistic? -Comprehensive quantitative research based on content analysis
Source: PLoS One. 2024 Mar 7;19(3):e0298601. doi: 10.1371/journal.pone.0298601 (PMC10919601; doi:10.1371/journal.pone.0298601)
Supplement: S1 Data — (ZIP) [file pone.0298601.s001.zip › Data/地级市Z_F工作报告.zip!/╜¡╬≈/╥╦┤║╩╨/└┤╫╘╣1⁄2╓┌║┼í╛┬φ┐╦╩2╛▌═°í┐_2009.docx]

各位代表：

　　现在，我代表市人民政府向大会作政府工作报告，请予审议，并请各位市政协委员和列席会议的同志提出意见。

一、2008年工作简要回顾

　　2008年是很不寻常、很不平凡的一年。在省委、省政府和市委的正确领导下，全市人民以开展“解放思想求突破、科学发展促赶超”主题教育实践活动为动力，深入贯彻落实科学发展观，积极应对来自各方面的严峻挑战和考验，众志成城，扎实工作，奋力拼搏，全面完成了市二届人大三次会议提出的主要任务。

　　“三类指标”同步提升。经济增长类、发展后劲类、民生保障类指标全面跃上新台阶，主要经济指标在全省排位基本实现“止后移”目标。经济增长类指标实现跨越。全市生产总值突破600亿元，达到615亿元，同比增长13.8%，增幅列全省第8位，比上年前移1位；财政总收入超过60亿元，达到64.9亿元，增长29%，增幅列全省第5位，比上年前移1位，地方财政收入36.5亿元，增长28.6%；丰城市财政总收入跃上15亿元新台阶，达到16.92亿元，樟树市超10亿元，高安市超7亿元，袁州区和上高县超5亿元，万载和奉新县超4亿元，丰城尚庄街办、高安八景镇超1亿元，实现了全市亿元乡镇（街道）零的突破；规模以上工业增加值达到212.9亿元，增长22.1%，增幅列全省第7位，比上年前移1位。发展后劲类指标快速增长。全社会固定资产投资突破300亿元，达到341.2亿元，增长41.6%；社会消费品零售总额190.4亿元，增长23.4%，增速创12年来新高；实际利用外资 2.94亿美元，增长10.1%；引进市外资金146亿元，增长12.2%；外贸出口2.85亿美元，增长34.9%。民生保障类指标有效落实。城镇居民人均可支配收入1.19万元，农民人均纯收入4683元，分别增长10.6%和13.8%；物价涨势得到控制，居民消费价格总水平上涨5.6个百分点；城镇登记失业率为3.2%；人口自然增长率控制在7.9‰以内。

　　“三化进程”明显加快。工业化强势推进，产业集聚效应日益凸显。签约引进重大项目106个，其中投资超5亿元的11个。9个工业园区入园企业1159家，完成主营业务收入512亿元，增长40.8%；税收27.7亿元，增长39.7%。科技入园深入推进，7个工业园被批准为省级民营科技工业园，“江西上高台商创业园”由省政府批准设立，奉新县争取到全省首个纺织印染集控区；省级产业基地增加到10个，高安建筑陶瓷基地被命名为“中国建筑陶瓷产业基地”。全市工业对经济增长的贡献率达64.2%，尤其带动了经济结构的优化和运行质量的提高，三次产业占GDP的比重由上年的22.2：50.3：27.5调整为21.7：52：26.3；财政总收入占GDP的比重达到10.6%，税收收入占财政总收入的比重达到90.8%，同比均提高0.7个百分点。城镇化加速提升，城市基础设施不断完善。全市城市建设投入达25亿元，村镇建设投入达23亿元，城镇化率达38.2%。各县市以城市创建为抓手，新建续建了一大批重点工程。重大项目建设取得突破，宜春明月山机场通过国务院立项审批，洪屏电站基本完成项目专题规划论证，武吉高速公路、320国道大万线、宜春至安福二级公路全面完工，高安至胡家坊一级公路实现通车，丰城剑邑大桥建成通车，曲江码头建设进展顺利，沪昆铁路客运专线正在规划设计，高安建陶基地铁路专用线已开工建设，樟树盐化工基地铁路专用线正在招投标。农业产业化稳步提高，“三农”工作再创佳绩。大力实施农业产业化“十百千”工程，全市国家级龙头企业由1家增加到4家，省级龙头企业由27家增加到44家。落实综合补贴3.9亿元，发放良种补贴1.04亿元、粮食直补资金1亿元、农机购置补贴资金2585万元。粮食连续5年增产，水稻总产再创新高。省级以上农业标准化示范区由7个增至16个。新农村建设扎实推进，落实试点建设财政配套资金1.36亿元，878个试点村平均投入30多万元，并全部成立新农村建设理事会，高标准建设100个示范点。农村基础设施建设继续加强，新修农村公路1308公里，新增通水泥（油）路行政村273个，新建沼气池1.2万个。争取中央及省水利投入5.67亿元，全国病险水库除险加固专项规划涉及的96座水库全部完成安全鉴定和初步设计，其中55座已开工建设，潦河、锦北、丰东、袁北四座大型灌区续建配套工程全面开工。17.3万人的饮水安全工程全面完成。农村渡改桥项目顺利实施。“万村千乡市场”工程稳步推进，丰城市列为全国“新农村商务信息服务工程”试点县。

“三大战役”深入推进。“工业强攻战”成绩喜人。宜春经济开发区投入建设资金1.72亿元，实施了东扩、立交桥涵洞、经发大道等工程，完成土石方1500万立方米，平整出可用土地6300亩；启动了污水处理厂、电镀集控区等工程，园区基础设施和配套能力逐步完善。新增入园企业38家，其中投资亿元以上12家，签约资金23.3亿元，实际进资6.1亿元；新增投产企业20家；实现财政总收入1.2亿元、主营业务收入26.9亿元、工业总产值20.6亿元，分别增长58.9%、140.9%、82.7%。初步形成了医药和医疗器械、汽车零部件、电子信息、机械制造四大主导产业。“城市扩张战”成果突出。宜阳新区总体规划基本完成，核心区建设全面展开，路网建设基本完成，19个重点项目完成投资7.5亿元。中心城区“13579”绿色工程深入推进，新建小游园11个、绿色文化广场3个、景观道路2条、公园1个，改造公园3个，7个公益林建设初具规模，种植8公分以上树木12.6万株。人口倍增计划有效实施，“五个一”便民工程顺利启动，中心城区户籍人口达31万，增加在校学生1.2万、三产从业人员1.2万，规划区外迁入人口1.3万，园区企业增加从业人员0.8万，中心城区人气渐旺。改造了宜阳大道、芦洲大道和明月北大道。高士北路延伸、秀江东路延伸、第五期小街小巷改造、明月大桥等工程建设顺利推进。沿河西路贯通、宜春南路连接、明月南路跨铁路立交桥工程的前期工作有效开展。污水收集管网一期工程全面竣工，污水处理厂一期续建工程稳步实施。供水一户一表改造扎实推进，天然气置换水煤气工作顺利完成。城市管理暗访综合评比连续四年列全省第一。“旅游升温战”成效显著。明月山温泉风景名胜区天工开物园、月亮湖、月亮湾文化园、月亮文化景点相继完成建设，启动了五星级酒店和梦月山庄主体工程，开通了上海—明月山旅游专列，开启了日本、韩国及香港“禅院行”旅游，开拓了上海、浙江两大新的旅游市场，首次接待一次性800人以上的旅游团队。以明月山风景名胜区为主体组建了市旅游集团。成功举办了第二届月亮文化节暨经贸活动周、香港等地的旅游推介活动。全年接待游客62万人次，增长24%；实现各类门票收入5100万元、旅游综合收入3.8亿元、财政收入3050万元，分别增长70%、81%和47%。

“三大环境”继续优化。围绕改革抓突破，创业环境进一步宽松。国有资产管理体制改革纵深推进，市直行政事业单位国有资产清收管理工作顺利展开。宜春汽运公司、宜工机械、煤气公司改制重组，长青公司依法破产进展顺利。启动了县级部门预算改革试点工作，国库集中收付制度改革全面推进。市直事业单位综合改革稳步实施。林权综合配套改革得到国家部委和省领导充分肯定，改革经验在全省推广。“农村义务教育债务监管系统”正式启用。78个国有农（林）场、农科所、良种场、畜牧水产场税费改革基本完成，“乡财县代管”全面完成。全民创业深入推进，汇编《支持全民创业和中小企业及非公有制经济发展优惠政策100条》，市直部门单位公开承诺服务创业的举措，试点启动全民创业园建设，探索建设中小企业信用担保体系。全市民营经济实现税金38.5亿元，增长22.2%。“优质服务年”活动扎实开展，开通了重大项目审批服务“绿色通道”。政府信息公开在全省率先上网开通，行政审批制度“两集中、两到位”改革全面展开，电子政务和公共服务信息化深入推进。全面调查清理了中心城区69家行业协会和61家市场中介机构。招投标交易活动进一步规范。制定实施了优化经济开发区外部环境“六个严禁”，严肃查处了公路“三乱”等一批破坏发展环境的典型案件。强化建管树品牌，生态环境进一步改善。坚持可持续发展，资源节约和环境保护得到加强。雨雪冰冻灾后完成补植补造28万亩，造林绿化“一大四小”工程全面启动。争取到国家投资土地整理项目3个，总投资达7233万元。出台了《关于加快生态文明建设的决定》，认真落实节能减排责任制，化学需氧量、二氧化硫等减排指标全面完成，水、大气、土壤等污染防治全面推进，宜春经济开发区成为首批省级创建生态工业园区试点单位。城市环境质量列全省第三。立足特色促繁荣，人文环境进一步浓厚。农村文化“111工程”稳步实施，农村和谐文化建设“一村一品”活动全面开展，工作经验向中宣部推荐。中国万载首届花炮文化节成功创办。文明城市和文明村镇创建工作力度加大。万载得胜鼓等6个项目入选国家级非物质文化遗产名录，靖安水口东周古墓被评为2007年中国十大考古新发现，高安华林周岭造纸作坊遗址在国内产生重大影响，宜丰天宝古村被评为中国历史文化名村。明月山国际华人华文作家笔会成功举办，《况钟明断十五贯》等一批优秀戏曲、影视和摄影作品获全国大奖。卫生服务能力和水平进一步提升，成为全省首个“全国农村中医工作先进市”，万载县创建省卫生县城通过考核鉴定。首届青少年科技小发明竞赛活动圆满成功，开全省青少年科普竞赛活动之先河。体育工作再创佳绩，杨文军在北京奥运会上蝉联男子划艇双人项目金牌，我市代表队赴法国参加《城市之间》国际版决赛，在10个国内参赛城市中名列第2。优化教育结构，加快普及高中阶段教育，普通高考二本线以上人数连续10年居全省前列，宜春职业技术学院顺利通过教育部高职高专院校人才培养工作水平评估。广播电视“村村通工程”完成率列全省第一，“广播村村响”试点工作积极开展。人口和计划生育工作平稳发展，出生人口素质得到提高，性别比偏高的问题得到有效控制，奉新县获全国计划生育优质服务先进县。

“三项保障”不断夯实。民生工程保障全面落实。全年发放小额贷款3.17亿元，解决困难群体“4050”人员就业10944人，零就业家庭就业安置率100%；城镇新增就业10.2万人，新增转移农村劳动力16.2万人。城、乡低保户月人均补助水平分别达到154.1元和56.3元，农村五保户集中供养率达到82.5%。城镇职工参加基本养老保险和基本医疗保险分别为51.4万人和62.8万人，其中国有困难企业职工参保23.6万人。全市参加新型农村合作医疗的农民达360.7万人。儿童一类疫苗全部实行免费接种。义务教育阶段的学生全部免除学杂费和免费提供教科书，4.4万名义务教育阶段家庭经济困难的寄宿学生、8491名普通高中贫困生、3万名中职学生和2016名考入大学的家庭经济困难学生得到政府资助。新开工建设经济适用住房16.6万平方米；采取住房实物配租和货币补贴相结合的方式，保障廉租住房受益家庭6861户，基本实现了保障对象的应保尽保。搬迁深山区移民2518人。农村基层残疾人组织建设工作经验得到中国残联的充分肯定。安全稳定保障扎实有力。深入排查调处矛盾纠纷，有效解决了一批群众反映强烈的急难问题，全市连续7年实现了“六无”目标。奥运期间，全市未发生1人次进京非正常上访，没有发生影响大局稳定的信访事件。全市公众安全感连续5年位居全省前列，宜春市被命名为全省唯一“平安市”。丰城市“转变作风赢民心，着力民生促稳定”的经验做法受到中央领导高度重视，并作出重要批示。持续开展安全生产专项整治，杜绝了工矿商贸企业重特大事故和火灾死亡事故，水上交通连续21年未发生死亡事故。食品药品、旅游等安全保持平稳态势，受“三鹿”奶粉事件影响的婴幼儿得到妥善治疗。人力资源保障富有成效。“6666”人才工程继续推进，人才队伍建设得到加强，评选了60名市政府特贴专业技术人才，选派了1000名科技特派员进村入户开展科技承包和农技推广活动，创办农业科技超市100多个，提供农技服务上万次。万载农村社会工作人才队伍建设模式在全国推广，丰城市培养大学生当“村官”的做法得到中组部充分肯定。3项科技成果获省科技进步奖，宜春学院“乌索酸标准样品”被批准为国家标准样品，填补了国内空白。

　　切实加强政府自身建设，加快推进政府职能转变和管理创新。坚持向人大报告政府工作，加强与政协联系，认真接受人大及其常委会的法律监督和政协的民主监督，89件人大代表建议、184件政协委员提案全部办复，满意率达97.8%。“五五”普法全面开展，法律援助和法律服务效能继续提升。廉政建设进一步加强，执法监察、廉政监察、效能监察和审计监督成效明显。国防后备力量和民兵预备役建设、人民防空、国家安全、外事侨务、民族宗教、涉台事务、气象服务、防震减灾、妇女儿童、统计、档案、扶贫、老龄和红十字会等各项工作都取得了新进步。

各位代表，回顾过去一年的工作，我们深感成绩的取得，是省委、省政府和市委正确领导的结果，是市人大依法监督、市政协民主监督以及社会各界大力支持的结果，是全市人民团结一致、共同奋斗的结果。在此，我谨代表市人民政府，向全市广大工人、农民、知识分子和干部，向各民主党派、工商联、无党派人士、各人民团体，向驻宜部队、武警官兵、公安干警、中央和省驻宜单位，致以崇高的敬意！向所有关心、支持宜春发展的离退休老同志和海内外朋友，表示衷心的感谢！

在看到成绩的同时，我们也清醒地认识到，经济社会发展中依然存在一些突出矛盾和问题，主要表现在：经济总量和增长速度与赶超式发展目标相比仍有差距；经济结构性矛盾较突出，产业结构优化升级的任务还很艰巨，经济增长的资源环境约束加大；城乡和区域发展不平衡，城市基础设施承载能力尚需提高，社会稳定的压力依然较大，突发事件应急处理能力有待加强，农业抗御自然风险和市场风险的能力较弱；社会就业压力依然较大，部分城乡居民生活困难，涉及群众切身利益的问题需加紧解决；改革攻坚的任务艰巨，政府职能转变和作风建设必须进一步加强，等等。对这些问题，我们将高度重视，积极努力地克服和解决。

　　二、2009年政府工作安排

　　做好今年的政府工作，意义与挑战都异常重大。在全球金融危机影响下的经济增速下滑，已在我市显现并将延续，实现全市赶超式发展的压力急剧加大。面对新形势、应对新考验，我们必须千方百计保障每一家生产企业的运转，千方百计促成每一个产业项目的落地，千方百计争取每一项重大投资的时效，千方百计抓住每一次赶超发展的机遇，千方百计激发每一份共克时艰的动力与活力，以创造性工作奋力实现保增长、扩内需、调结构、利民生的目标。

　　今年政府工作的总体要求是：全面贯彻胡锦涛总书记在江西考察工作时重要讲话和省委、省政府工作部署及市委二届六次全会精神，以邓小平理论和“三个代表”重要思想为指导，以开展科学发展观学习实践活动为契机，坚持赶超式发展不动摇，着力推进大项目、大产业建设和固定资产投资较快增长，着力推进工业化、城市化和农业产业化进程，着力深化重点领域改革和提高对外开放水平，举全市之力，聚全民之智，解放思想，求真务实，迎难而进，化挑战为机遇，变危机为生机，确保实现经济平稳较快发展和社会全面进步，以优异的成绩迎接新中国成立60周年。

　　综合考虑各种因素，今年国民经济和社会发展主要目标建议为：生产总值增长12%；财政总收入增长20%，地方财政收入增长18%；规模以上工业增加值增长25%；全社会固定资产投资增长35%；社会消费品零售总额增长18%；引进省外5000万元以上工业项目资金增长20%；实际利用外资增长10%；外贸出口增长20%；农民人均纯收入增长8%，城镇居民人均可支配收入增长8%；万元生产总值能耗下降4.5%，万元工业增加值能耗下降5%，化学需氧量排放量下降2.1%，二氧化硫排放量下降2.4%；城镇登记失业率控制在4.5%以内；人口自然增长率控制在8‰以内。

　　为实现上述目标，重点抓好以下五方面的工作：

　　（一）加速推进大产业、大项目建设，大幅度增加项目投资规模

　　全力推进重大项目建设，增强经济发展后劲。紧紧抓住中央扩大内需、加大投入的机遇，把项目建设作为经济工作的重中之重、急中之急。按照“加快推进一批项目、积极跟进一批项目，精心策划一批项目”的总体要求，认真研究政策，及时掌握信息，密切跟踪联系，全力以赴做好向中央和省申报项目工作，力争我市项目资金占全省的份额不少于8%。抓紧对已批重大项目的实施，开工建设宜春明月山机场、洪屏电站、“杭南长”高速铁路、奉新（靖安）至南昌高速公路等重大基础设施项目，全面启动交通部特别补助的县至乡399公里公路建设项目。继续抓好高安建筑陶瓷基地和樟树盐化工基地铁路专用线、宜春铁路货场改造等项目建设，抓紧做好九江—长沙铁路、武汉—吉安铁路、丰城铁路专用线、上高电厂等重大项目前期工作，争取早日开工建设，早日发挥效益。进一步完善项目推进机制，落实工作责任，对市领导挂点的86个重大项目加强调度，严格考核，确保进度。

　　全力加速产业基地建设，形成规模聚集效应。深入开展十大产业基地“产业上规模，项目上质量，效益上台阶，信用上等级，基础建设上水平”创评活动。进一步完善产业规划和产业配套，搞好产业链设计和项目策划，瞄准国内外优强企业和科研院校，实行针对性产业招商，促成一批投资规模大、科技含量高、创税能力强的项目落户。抓紧丰电三期超临界发电机组扩建项目立项，加快年产220万吨高焦化项目、年产90万吨原煤的丰龙矿井项目、凯迈非晶硅、盛华多晶硅、斯米克、东鹏、唯美及8个陶瓷配套项目建设。做大做强奉新金源纺织、宝源彩纺一期、三邦线业、赣鑫纺织二期。促进樟树蓝恒达、冀鲁化工、国宏化工、通能硅材料顺利达产，抓紧四特酒厂扩建、仁和集团二期工程和瑞祥化工建设，积极跟进三友集团80万吨联碱项目和湖南五江轻化循环项目。力促高安建筑陶瓷基地已投产生产线满负荷生产，确保在建生产线全部竣工投产，力争上半年新明珠、新中源各新增投产生产线8条。加快上高裕盛鞋业二期及宜丰、宜春基地新增18条生产线的建设，抓紧匹克鞋业鞋底厂、发泡厂和2条成型线建设。重点支持袁州济民药业金水宝胶囊二期技改项目建设，帮助百神集团新上“克黄利胆胶囊”和“2046”等生物药品二期生产线，加快远大生化、梵福民生物科技、博世强、长征医药等项目建设。加快推进市本级江特电机“低压大功率变频调速三相异步电动机”项目建设，加快业宏机床建设步伐，帮助海佳电器开拓市场。扶持靖安天合新材料有限公司一期项目尽快达产达标，努力引进6家投资5000万元以上相关项目入园。创新金融产品，着力破解企业融资难题。整合国有资产，尽快构建全市统一的融资平台，扩大注资规模，增强担保实力。认真抓好小额贷款公司试点，加大异地融资力度，在加强信贷合作的基础上，争取更多的外埠金融机构入驻宜春。提高入园企业土地利用率，严格节约用地制度，依法依规做好清理闲置土地工作。

　　 全力提升招商引资水平，促进开放型经济发展。坚持把招商引资作为促进大投入、建设大项目、形成大产业的首要举措，深入开展“重大产业招商推进年”活动，重点围绕全市十大产业基地，组织精干团队，开展产业招商。同时围绕我市优势资源和产业基础，紧盯国外500强、国内200强企业，突出“长珠闽”、港澳台、日韩欧美等重点地区、重点区域，针对性地开展大项目招商。进一步改进招商方式，注重开展小分队招商、专业招商、以商招商、以亲招商。努力优化外贸主体结构和出口产品结构，着力引进生产型出口企业，培育壮大一批出口支柱企业，力争全市年出口上千万美元企业4个、500万美元以上企业10个。积极组织有条件、有意向的企业参加会展、考察活动，引导扶持有实力的企业到境外设点办厂，参与国际经济竞争与合作。强化推进口岸通道建设，争取设立宜春海关办事处，上高鞋革及节能灯、万载有机农产品、丰城富硒农产品、高安建筑陶瓷检测中心，积极筹建县（市）外汇管理分支机构，努力降低企业商务成本。

　　 全力拓展“三大战役”成果，增强中心城区辐射力。围绕打造“中部最佳宜居城市”目标，继续深入推进“三大战役”。按照宜春经济开发区产业发展规划及市本级6大产业建设目标，大力实施产业招商，实现工业企业主营业务收入30亿元，财政收入1.8亿元。进一步突出园区省级机电产业基地特色，力争四大主导产业销售收入在园区中的比例提高到60%以上，引进重大项目6个、新批外资项目2个。进一步落实领导挂点责任制，确保宜春碧彩、新瑞泰、宾利汽车电器、恒珠柜锁等企业建成投产。抓紧东扩工程建设，不断完善园区基础设施。根据宜阳新区发展规划及人口倍增计划、“13579绿色工程”建设要求，抓紧宜阳新区核心区建设项目的进度，尽快形成新区的建设形象和带动功能；推进单位自建办公项目、宜阳安置小区及中心城区防洪工程、三级抬水坝等在建重点工程项目建设。加快启动机场路A线和B线、明月南路拉通（含明月南路跨铁路立交桥）、芦洲大道南延、宜春南路贯通、环城西路320国道改造、秀江东路至下浦延伸等六条道路建设。快速推进服务业发展，规范提升传统服务业，有效发展现代服务业，进一步加大招商引资力度，开工建设五金家居商贸城和中国（南方）农机大市场项目，争取宜春迎宾馆、城标广场五星级酒店、房地产交易大市场以及时尚消费等一批重点项目早日开工建设，积极扶持赣西农产品市场做大做强。围绕打造月亮之都、禅宗之地、宜居之城旅游品牌特色，抓好旅游发展规划，整合全市旅游资源，精心打造并推介月亮文化、禅宗圣地、古色人文、红色故土、绿色山水五条旅游精品线路，启动禅都文化博览园和秀江花月夜景观带建设，做好景区与中心城区互动并向县（市）延伸的文章，进一步巩固和开拓国内外旅游市场。抓好市旅游集团公司的市场化运作，创建一批A级景区、星级酒店、品牌旅游产品，力求在打响旅游品牌、加快旅游设施及要素建设上取得明显成效。精心筹办第三届月亮文化节暨经贸活动周。抓好项目建设，力争中心城区有3个以上、各县（市）城区至少有1个3000万元以上的旅游开发项目开工建设。大力推进明月山景区建设，打通温汤片区与洪江片区的快捷通道，建好温汤至古庙旅游公路、温汤环城路、里布水库、青云栈道，启动温汤集镇改造和温汤河整治工程，建好明月山顶至十八排电瓶车道，加快推进国际温泉禅修中心、五星级酒店等景区娱乐、宾招购物设施建设。力争全市接待旅游总人数增长20%；旅游总收入增长28%。

　　（二）进一步强化农业基础地位，努力再创“三农”工作新优势

　　 稳步提高农业产业化经营水平。重点推进粮食、竹木、家禽、中药材、油茶、生猪、肉牛、苎麻、果业、有机及富硒农业等十个优势产业和特色产业进程。积极策应全省“新增百亿斤优质商品粮生产能力工程”建设，保持全市粮食生产稳定增长，不断提高优质水稻生产比重，积极发展粮食产品精深加工，做响玉珠、圣牛、汇银等粮食名牌，做强宜春天地粮食集团、江西金农米业集团。认真组织实施好100万亩高产油茶林基地建设项目，力争全市新造高产油茶林6万亩，改造低产林10万亩。以万载、铜鼓、宜丰为重点，着力做大基地，做强龙头，做出品牌，力争新增通过认证基地10万亩，力争建成江南最大的有机农产品生产基地；以丰城生态硒谷科技园为依托，加快建设特色鲜明的富硒农产品生产基地。大力发展规模养殖，强化生猪品种改良，提升精深加工水平，带动全市生猪产业结构的整体升级，力争全市出栏生猪50头以上的养殖户达到1.2万户以上，全市省级种畜禽场发展到10家以上。努力引进和培育农业产业化龙头企业，每个县（市、区）新上1个以上投资规模1亿元以上的农业产业化龙头企业和产业化项目，力争涉农产业招商引资额占全市招商引资总额的20%以上。鼓励发展农民专业合作社，重点扶持 “一村一品”专业村100个，新增各类农民专业合作社100家。

　　大力推进新农村建设。在抓好产业发展的同时，突出村庄整治和“三绿一处理”建设。在着力解决好“走平坦路、喝干净水、上卫生厕、住整洁房、用洁净能源”等问题的基础上，按照绿化净化美化的要求，推进新农村点庭院绿化、道路绿化、村旁绿化和垃圾无害化处理，努力打造一批房前屋后花果园、村内道路林荫化、村庄周围风景林的生态文明村，使村庄人居环境和生态环境得到明显改善。积极鼓励生态县市、环境优美乡镇和生态村建设，把生态村建设和新农村建设有机结合起来。突出农村新社区建设。积极开展创业文化、合作文化、科学文化、道德文化、法制文化建设活动，组织实施“新农村新家庭计划”，培育新型农民；健全村民自治机制，完善村民理事会功能，积极开展群众性文化体育活动，促进乡风文明和管理民主。在抓好1000个左右新农村点的基础上，实施新农村建设和谐小康家园示范工程，突出抓好100个示范点建设。继续实行市、县两级领导、部门、企事业单位挂点帮扶制度，建立和完善帮扶新农村点建设的长效机制。

　　 继续加强农业基础建设。认真组织实施优质粮食、大中型沼气建设、国家现代林业示范、高产油茶基地、病险水库除险加固、大型灌区续建配套与节水改造、农村饮水安全、小型农田水利建设、土地整理、农业综合开发等10大农业工程项目，不断改善农业基础条件。积极引导土地承包经营权流转，推进适度规模经营，提高土地产出率、资源利用率和劳动生产率。大力实施“造地增粮富民工程”，全面推进土地整理开发，确保耕地占补平衡。巩固并深化林权配套改革成果，全面启动和推进现代林业示范市建设，继续抓好生态公益林、退耕还林、长防林工程建设，实施封山育林，严厉打击乱砍滥伐行为，突出抓好国家和省级自然保护区、森林公园建设，推进造林绿化“一大四小”工程，确保全市造林绿化58.5万亩。加快乡镇政府职能转变，完善社会管理和公共服务，全面推进和规范乡镇“便民服务中心”建设及农业技术推广体系的试点和建设。加强农产品质量安全体系建设，切实抓好产地安全检测工作，严防重大动物疫病和农产品质量安全事故发生。

（三）积极深化体制机制的改革与创新，不断激发赶超发展的内在动力和活力

　　继续深化各项改革。强化国有资产监管，推进投融资体制改革，完成市直行政事业单位国有资产清收工作，分类整合市直行政事业单位国有资产，充分发挥新型投融资平台及资本运作平台作用。积极探索无形资产及资源性资产监管途径。深化国有企业改革，基本完成市属企业的改革改制及政策性破产，促进资产重组和新的发展。继续推进企业上市工作，力争年内华伍制动成功上市，帮助百神医药、南特公司、远大化工等企业完成上市准备。完善招投标管理体制改革，整合资源打造集中统一的招投标有形市场，营造公开、公平、公正、有序的招投标市场竞争环境。深化财税管理制度改革，健全公共财政体制，完善部门综合预算，将行政事业性收费全面纳入预算管理，着力建立以绩效为导向的部门预算编制模式，积极稳妥开展公务卡制度试点，认真执行新修订的增值税、营业税、消费税暂行条例，全面实施增值税转型改革。继续深化人事制度改革，加快事业单位岗位设置管理实施工作，积极推进事业单位人员聘用制，深化职称制度改革。按照省政府统一部署，积极推进地方机构改革。理顺和规范中心城区公安机构设置，有效整合和充分利用警力资源，建立指挥顺畅、权责明晰、运转协调、实战高效的警务运行机制。冷静应对房地产发展的严峻形势，落实配套优惠政策，适当放宽住房信贷政策，进一步鼓励和支持普通商品住房消费，保持合理的房地产开发投资规模，促进房地产市场健康发展。

　　进一步优化发展环境。以提高效率、优化服务为重点，全面落实推进工业化、城市化和发展民营经济各项优惠政策，全面落实市直单位支持开发区发展的各项政策措施，努力把宜春打造成为全省乃至中部地区最开明、最诚信、最务实、最高效的地区。严格执行优化开发区发展环境“六条禁令”，全面畅通“绿色通道”服务，建立重大项目窗口跟踪服务联系制度。继续巩固和深化市县两级行政审批制度“两集中、两到位”改革，目前保留的行政许可事项、投资项目审批事项、单项行政审批时间在现有承诺的时限基础上均完成缩减30%的目标，全面推行“一站式”办证、“一单清”审批、“一条龙”服务，对重点项目全面实行办证办事代理制。进一步抓好市县两级中心的网上行政审批和电子监察系统建设。认真开展“治‘三乱’、减负担、优环境”专项活动，切实为企业减负。继续对各类学会、协会、中介机构进行清理整顿，规范收费行为。完善环境监测和效能监察工作机制，认真执行问题协调联动和投诉处理快报机制，积极推动建立多渠道社会评议评价体系，加大综合查处力度，对破坏发展环境的典型案件进行曝光。

（四）不断深化民生工程建设，努力促进社会事业协调发展及和谐稳定文明环境创建

　　精心实施好以就业为重点的民生工程。把保障和改善人民群众冷暖安危作为重要职责，按照“量力而行、尽力而为、统筹兼顾、可持续实施”的要求推进各项民生工程建设。突出抓好就业再就业工作，大力促进大学生就业，认真抓好失业人员、就业弱势群体、农村富余劳动力、退役士兵和高考落榜生培训和就业；尤其要迅速开展返乡务工人员与本地企业的对接工作，有效化解就业难与招工难的矛盾，促进返乡就业与创业。加快建立和完善以签订劳动合同为基础的劳动用工备案制度，提高劳动合同签订率。加大社会保险扩面征缴力度，不断完善社会保障体系，提高社会救助水平。适时调整、提高城乡居民最低生活保障标准。提高农村五保对象的供养标准和管理服务水平，确保集中供养率稳定在80%以上。确保企业离退休人员养老金按时足额发放，社会化发放率和支付率均达到100%。完善城镇职工、居民基本医疗保险和城乡困难群众医疗救助制度，逐步提高医疗保障水平。大力加强医疗保障和食品药品安全综合监管体系建设，加强公共卫生应急体系建设，提高防范和处置公共卫生事件的能力，建立健全医患处置机制，强化部门协作机制，确保食品药品安全。加大保障性住房建设力度，扩大廉租住房保障范围，加大廉租住房实物配租力度，拓宽经济适用住房建设思路，加大城市危旧房改造力度，不断改善市民居住条件。推进县市各乡镇住房公积金制度的建立。实施好“阳光工程”、“雨露计划”整村推进、移民搬迁、农村饮水、户用沼气建设、农村公路、农村困难群众住房维修改造等工程。

　　推进各项社会事业全面进步。大力实施人才强市战略，切实加强公务员、专业技术人才、企业管理人才、农村实用人才和社会人才队伍建设。完善教育布局，优化教育结构，提高教育质量。推进教育“三名”工程，全面实施“5611”计划，即用五年的时间在全市中小学及幼儿园重点培养名师600名、创建高中阶段教育名校10所、培养名校长100名。重视发展高等教育工作，不断提升办学质量与规模。继续推进中小学、幼儿园新校建设，努力改善办学条件，切实解决大班额问题。优化科技资源配置，促进科技成果向现实生产力转化。以科技大开放为先导，着力抓好科技项目建设，积极实施科技入园、科技进村和科技惠民工程，注重培养一线科技创新人才，加快重大科技成果产业化步伐。加快信息化建设，积极支持和推进电子政务、电子商务以及TD等3G网络建设运营工作。进一步发展文化事业。办好第三届宜春市文化艺术节，突出抓好禅宗遗址保护及开发为基础的禅宗文化，以非物质文化遗产保护及开发为基础的传统地方文化，以文化演艺业为主体的娱乐文化，大力实施农村“广播村村响”、农村“一村一品”特色文化建设、数字电视平移、文化信息资源共享、农村数字电影放映、农家书屋工程。加大卫生服务能力建设。巩固农村中医工作、加强公共卫生服务能力建设、扩大卫生服务覆盖面，加快卫生品牌建设工程，着力培养名医、构建名科、打造名院。广泛开展全民健身活动，促进竞技体育和体育产业发展，办好市第三届运动会。加强生态文明建设，依法保护和合理开发生态资源，淘汰和关闭一批高耗能、高污染、低产能和低水平重复建设的企业。坚持最严格的耕地保护制度。做好节能减排工作，重点加强对城市污水处理厂建设的环保指导和服务。严把建设项目环保准入关，坚持从源头控制污染，加大农村面源污染的治理力度，积极开发利用可再生资源，加快循环经济发展。进一步做好人口和计划生育工作，完善人口和计划生育利益导向机制，稳定低生育水平，巩固出生人口性别比失调整治成果，提高出生人口素质。

　　狠抓安全生产和社会稳定。继续抓好煤矿、烟花爆竹、非煤矿山、危险化学品、道路、水上交通等专项安全整治，狠抓森林防火工作和公众聚集场所的消防安全。进一步强化企业主体责任和安全监管责任，建立健全隐患排查治理长效机制，严厉打击违法违规行为，杜绝重大以上安全事故发生。规范行业安全管理，减少非正常死亡事故。依法防范和坚决打击各种刑事和经济犯罪活动，规范经济秩序，保障人民群众生命财产安全。在市、县中心城区组织开展以打击侵财性和暴力犯罪为重点的专项行动，在农村地区组织开展集中打击整治行动和重点“治乱”工作。做好矛盾纠纷排查调处工作，重点排查调处征地拆迁、资源权属、企业改制、社会福利、劳资关系以及食品卫生等涉及民生利益的矛盾纠纷，积极预防和坚决防止发生重大群体性事件。建立健全矛盾纠纷解决机制，不断推进人民调解、司法调解、行政调解“三调联动”。进一步加大法律援助工作力度。注重教育和引导群众以理性和合法的方式表达利益诉求，认真办理群众通过网上信访、电话、短信、绿色邮政等渠道反映的诉求和建议，充分发挥市、县两级人民来访接待中心功能，拓宽群众信访渠道。重视国防教育，提高民兵预备役建设质量，搞好国防动员。

（五）强力推进政府自身建设和“机关效能年”活动，进一步提升依法行政和廉洁从政效率

　　发展形势的新变化和人民群众的新期待，对政府工作提出了新的更高要求。我们将以自我加压、负重奋进的精神，更好地承担起赶超式发展的历史重任和光荣使命，倾心尽责地对人民负责，受人民监督，让人民满意。

　　进一步增强责任意识和操作能力，更加注重科学发展。按照市委统一部署，扎实开展好科学发展观主题教育实践活动，切实将学习实践活动与加强领导班子思想政治和干部队伍建设结合起来，与应对危机、克服困难结合起来，坚定不移地把科学发展观的要求贯穿于经济社会发展的各个方面，不断提高科学判断形势的能力、驾驭市场经济的能力、应对复杂局面的能力和总揽全局的能力。各级政府及其工作人员，务必把生机大于危机的理念，变成赶超式发展的实际行动，以新的作为应对新的挑战和考验。要牢牢把握大局，敢于担当责任、敢于承担风险，善于从国内外形势发展变化中把握发展机遇，善于从新一轮赶超式发展实践中创新发展方式、破解发展难题，着力解决影响和制约科学发展的突出问题，着力解决人民群众最关心、最直接、最现实的利益问题，做到发展为了人民、发展依靠人民、发展成果与人民共享。

　　进一步改进政府职能与机关作风，更加注重行政效能。进一步理顺政府与企业、市场、社会的关系，在加强和改善经济调节、市场监管的同时，更加注重社会管理和公共服务，维护社会公正和社会秩序，促进基本公共服务均等化。加强和改进对基层、对群众、对社会的服务。在全市认真组织开展“机关效能年”活动，深入推进政府信息公开，努力提高行政效率和公共服务水平。大力弘扬求真务实，以务实促发展，最大限度地减少会议、应酬、检查和评比，进一步形成快捷、朴实的文风政风，树立讲认真、强督导、抓落实的工作作风。各级政府领导干部都要切实做到重落实、重操作，按照“项目化、时间表、责任人”的工作要求，加强工作调度和督查考核。对重要工作定期调度、定期通报，跟踪问效、督促落实，严格考核、兑现奖惩，确保每一项重大政策、重要举措、重点工程顺利实施，取得实效。坚持依法行政，进一步推进政府行为的法治化、程序化、规范化。自觉接受人大及其常委会的依法监督、人民政协的民主监督，及时办理人大代表建议和政协委员提案，广泛听取各民主党派、工商联和无党派人士的意见和建议。健全社情民意反映制度和重大事项专家咨询、社会公示、听证和质询制度，切实做到科学决策、民主决策。

　　进一步强化节俭约束与廉政防线，更加注重队伍建设。各级政府务必努力树立六种形象：比奉献，树为民形象；勤学习，树理性形象；正品行，树正派形象；讲民主，树团结形象；重实干，树务实形象；守纪律，树清廉形象。要增强党性观念，不断改造世界观、人生观和价值观。要坚持贯彻民主集中制，健全并严格执行民主集中制的各项规定，切实维护领导班子的团结。要身体力行，深入一线，勇于奉献，在实践中建功立业，在实干中赢得认可。要清正廉洁，在国家加大投入、扩大内需的政策拉动下，今年市里的项目多、投资大、工期紧，加强项目资金监管、防止腐败发生是今年廉政建设的重点。各级领导干部要严格遵守有关规定，筑牢拒腐防变的思想道德防线。进一步落实党风廉政建设责任制，以党员领导干部为重点，找准腐败问题易发多发的重点领域和关键环节，加强对权力部门和领导干部的监督，认真查处腐败案件和严重侵害群众利益的典型案件。进一步强化过紧日子的思想和约束，严格实行公务购车经费、会议经费、公务接待经费、党政机关出国（境）经费预算“四个零增长”。继续抓好“六条禁令”的贯彻落实，严格规范公职人员的从政行为，努力在人民群众中树立良好形象。

　　各位代表！在新的形势、新的考验中，我们肩负的宜春赶超式发展重任崇高而艰巨。让我们高举中国特色社会主义伟大旗帜，在省委、省政府和市委的坚强领导下，坚定信心，开拓进取，奋力拼搏，以全市经济平稳较快发展和社会全面进步的优异成绩向新中国成立60周年献礼！
